# What are the factors associated with rural-urban inequality in under-5 deaths in low- and middle-income countries? A Fairlie decomposition analysis

**DOI:** 10.1371/journal.pgph.0004394

**Published:** 2025-04-09

**Authors:** Adeniyi Francis Fagbamigbe, Taiwo Akinyode Obembe, Oyewale Mayowa Morakinyo

**Affiliations:** 1 Department of Epidemiology and Medical Statistics, Faculty of Public Health, College of Medicine, University of Ibadan, Ibadan, Nigeria; 2 Department of Health Policy and Management, Faculty of Public Health, College of Medicine, University of Ibadan, Ibadan, Nigeria; 3 Department of Environmental Health Sciences, Faculty of Public Health, College of Medicine, University of Ibadan, Ibadan, Nigeria; African Population and Health Research Center, KENYA

## Abstract

**Background:**

The retention of under-5 mortality (U5M) in various ramifications has dire policy implications. The varying impacts of this inequality is very important and has been researched in many rural-urban settings. In spite of many studies that have examined rural-urban inequalities, very little has been researched with respect to low middle-income countries. In this study, we utilized an innovative statistical method to examine and explain the socio-economic determinants and rural-urban differences of mortality in some selected low- and middle-income (LMIC) countries.

**Methods:**

Using secondary data from the Demographic Health Survey (DHS), we utilized a Fairlie decomposition analysis to enumerate the differences amongst under-5 populations across 59 low-middle income countries in four continents. Death of any child within 0 – 59 months of life was our dependent variable while some selected individual and neighborhood factors constituted the explanatory variables.

**Results:**

Study findings revealed significant pro-rural and pro-non-rural inequities across the 59 countries. Pro-rural inequities were more commonly found in the African regions. Except for the Maldives, pro-non-rural inequities were largely associated in the remaining four continents. Some factors, unemployed status, ever married or single status, female household head, insurance cover, unimproved water sources, clean fuel were associated with a higher risk of Under-5 mortality.

**Conclusion:**

The results from this study are pertinent to health system reforms needed to tackle the menace of under-5 mortalities in LMICs and worldwide. Consolidation of existing maternal and child health programs supported by a resolute and firm re-evaluation of political will considerably help to control the surge of U5MR in the countries studied.

## Background

The under-5 mortality rate (U5MR) is the probability of death occurring before the age of five years per 1,000 newborns [1,2]. Mortality in children has always been a priority concern for decades [3]. So much so that it forms a major component and critical indicator in both the defunct] Millennium Development Goal 4 (MDG-4) and the United Nations Sustainable Development Goal 3 (SDG-3.2.1) [4,5]. The under-5 mortality rate is an important indicator in any health system because it reveals the level of economic, social, environmental and health care conditions that children are subjected to and ultimately the relative child survival probability for every child below the age of five years [2].

Despite the recommendation by the Programme of Action of the International Conference on Population and Development (ICPD) that advocates attainment of 45 deaths per 1000 live births among under-five children by 2015 for all countries [1], literature affirms that an estimated 70 million children die before 5 years of age. Worse still, children residing in low-middle income countries are ten times more likely to die compared to children in high-income countries [6]. Even though worldwide under-5 mortality rates have witnessed a reduction from 12.6 million in 1990 to 5.2 million in 2019 [7], Northern Africa and Eastern Asia have experienced much higher reductions in under-5 mortality rates (68% and 70% respectively) when compared to sub-Saharan Africa that has only been able to bring down its U5MR by only 39% [8–10].

The Sustainable Development Goals (SDG 3.2.1) aims to end all preventable deaths of newborns and children under 5 to as low as 25 per 1,000 live births by the year 2030 [5,11]. Some countries have met the target while others are still far from it. Reports from World Data estimate the U5MR in the USA to be as low as 6.98 deaths per 1000 live births while countries such as Nigeria still have rates as high as 95.12 deaths per thousand live births in 2020 [12].

Literature has documented a higher preponderance of childhood deaths among children raised in rural areas than in urban areas. These differences in U5MR are particularly pronounced in rural regions compared to urban regions. In a south-central Asian province, the magnitude of the under-5 mortality was approximately 3 times higher in rural than in urban Bhutan [13]. In sub-Saharan Africa particularly, the magnitude between the rural-urban dimensions in under-5 mortality has been significant. Although Van de Poel et al. and emphasize the need to consider household and community characteristics in understanding mortalities [14], Van Malderen and colleagues extend the need to also consider individual factors in addition to household and community factors [15].

Documented factors influencing under-5 mortality rates in sub-Saharan Africa range from health, environmental to maternal socioeconomic and demographic characteristics factors such as the age of mother at first birth, the age of mother at the time of under-5 mortality occurred, ethnicity, number of household members (3-5children and ≥6 children versus 1-2 children), polygyny vs monogyny, household access to electricity, region, maternal education, sex of household head, wealth index, mother residing with husband/partner at the time of under-5 mortality occurred [3,16–19]. Some studies have equally explored deeper underlying factors that influence under-5 mortality across various regions. For instance, Yaya et al. decomposed the contributions of associated factors to rural-urban differentials under-5 deaths across sub-Sahara African countries [20]. Likewise, Van De Poel et al. explored variations in under-5 mortality among six Francophone countries in Central and West sub-Saharan Africa [14]. In another recent study by Fagbamigbe and colleagues, even though the interest was also among under-5 children, the focus centred on socioeconomic determinants of educational inequalities in diarrhoea [21]. Harttgen et al. also conducted a multi-level country analysis of under-5 mortality between 35 sub-Saharan African countries and 13 Asian countries from 1992 to 2016 [22]. However, none of these studies explored rural-urban inequalities in under-5 mortalities across the low- and middle-income countries (LMIC). Furthermore, their study focused on only countries within two geographic regions (Africa and Asia) alone [22].

To ensure a continuous and critical analysis of rural-urban differences that is crucial to understanding the economic and social development of developing countries [23], a synthesis of rural-urban differences in U5MR across the globe is imperative to provide robust and relevant information needed for global under-5 mortality interventions. In this study, we explore rural-urban differences across 6 geographical regions (America, Africa, Asia, Europe, Caribbean and Oceania) using the Fairlie decomposition method. The results from this study will not only add to the scientific body of knowledge but also provide important findings needed for policymakers across the 6 regions to proffer policy initiatives that contribute towards the closure of rural-urban gaps and inequities in selected countries [24]. To the best of our knowledge, this is the first study that identifies factors contributing to rural-urban inequalities in under-5 mortalities across six continents.

## Methods

The data sources for this analysis are all Demographic and Health Surveys (DHS). The DHS holds a partnership led by ICF in the USA in conjunction with the designated organizations in the participating LMIC every five years. DHS are cross-sectional, nationally representative and population-based. The data have been pooled and merged into a dataset of 59 most recent nationally representative household surveys from 59 LMIC conducted between 2010 and 2018 and released as of September 10th, 2020. In all, 856,987 under-5 children living in 66,495 neighborhoods nested in 59 LMIC were included in this study.

Over the years, DHS adopted clustered multi-stage sampling technique across each of the participating countries. The number of clusters in each country depended on the prevailing geographical and administrative structures in the country. The multi-stage procedures use the states/divisions/regions in the first stage, districts as the next stage in some countries and the clusters are selected at the last stage. The clusters are the primary sampling units (PSU) [25,26]. The households were then selected from the PSUs. The most recent census of each country was used as the sampling frame. Typically, members of the population have unequal chances of being selected as a result of the clustered sampling. Sampling weights were computed and provided alongside the data by DHS to correct the unequal chances of sampling. A similar set of survey and research protocols, standardized questionnaires, training of enumerators, supervision, and implementation were adopted in all the countries. Further details of the sampling methodologies and other information are available at dhsprogram.com [27].

The outcome variable children survival within the first five years of life is commonly referred to as “under-5 death”. Under-5 death is death within 0 to 59 months of birth [28]. Mothers were asked to recall all children they had within five years preceding the survey. The mothers then provided the living status (alive or dead) of each of the children. Therefore, “under-5 death” has binary outcomes: “*Alive as of 5*^*th*^ *birthday” or “dead before 5*^*th*^ *birthday*”.

The rural-non-rural (rural-urban) differentials in the location of the residence of the mothers are the main determinate variable in this study. The DHS used similar standard classification procedures as of the time of the surveys with minimal differences in what rural areas were across the countries. These classifications were embedded in the dataset.

In addition to the identified variables in the literature [14,16–18], Moseley’s systematic conceptual framework on the study of child survival in developing countries was used to select the explanatory variables in this study [29]. The independent variables used in the study are the individual-level and neighborhood-level factors identified in the literature to be associated with childhood deaths.

The individual-level factors are weight at birth (average+, small and very small), birth interval (firstborn, <36 months and>=36 months), sex of the child (male or non-rural), birth order (1, 2, 3 and 4+), a child is a twin (single, multiple (2+)), maternal education (none, primary or secondary plus), maternal age (15 to 24, 25 to 34, 35 to 49 years), marital status (never, currently and formerly married), maternal and paternal employment status (working or not working), health insurance (yes/no), sex of the head of the household (male or non-rural), access to media (at least one of radio, television or newspaper), sources of drinking water (improved or unimproved), toilet type (improved or unimproved), cooking fuel (clean fuel or biomass), housing materials (improved or unimproved) and household wealth index (poorest, poorer, middle, richer and richest).

We used “neighborhood” to depict the clustering of children. The “clusters” are the PSUs. People of the same cluster that shares similar contextual factors [25,26]. We computed neighborhood socioeconomic status (SES) as a neighborhood-level variable. It was aggregated from the proportion of mothers within the same clusters without education, belonging to a household in the two lowest wealth quintiles, no media access and unemployed using the principal component factor method.

Descriptive statistics and inferential statistics were used. The descriptive statistics include charts, tables, and percentages. Bivariable analysis was conducted using the Z-test for equality of proportions of under-5 deaths among rural and non-rural children within each country and region (Table 1). The distribution of the explanatory variables and the outcome variable among rural and non-rural children were presented in Table 2. The risk difference (RD) in under-5 deaths among rural and non-rural children were computed. A RD > 0 implied a higher risk of under-5 deaths among rural children than among non-rural children (pro-rural inequality). While a RD = 0 signifies no difference, a negative RD (RD < 0) indicates that under-5 deaths are higher among non-rural children than among rural children (pro-non-rural inequality).

**Table 1:** Distribution of sample characteristics by countries, regions, and prevalence of under-5 deaths in LMIC by rural-urban differentials in place of residence, 2010–2018.

Country	Year	Sample	Communities	Rural Rate	Under-5 Deaths per 1000 livebirth		
					Rural	Non-rural	Overall
Overall		856,987	66495	67.7	51	57	^*^40
Eastern Africa		109,945	6298	78.9	52	53	51
Burundi	2011	13,192	554	90.9	59	59	52
Comoros	2012	3,149	252	73.2	42	48	^*^26
Ethiopia	2016	10,641	643	89.0	55	56	^*^43
Kenya	2014	20,964	1593	64.1	44	41	^*^49
Malawi	2016	17,286	850	86.7	49	49	46
Mozambique	2011	11,102	610	72.3	74	76	70
Rwanda	2014	7,856	492	83.2	39	40	33
Tanzania	2015	10,233	608	72.9	53	47	^*^68
Uganda	2016	15,522	696	78.8	51	53	^*^43
Middle Africa		76,790	2932	57.8	70	79	^*^59
Angola	2016	14,322	625	39.6	51	64	^*^43
Cameroon	2018	9,733	429	55.3	62	69	^*^52
Chad	2015	18,623	624	80.3	98	97	102
Congo	2012	9,329	384	39.4	51	48	54
Congo DR	2014	18,716	536	69.4	75	78	^*^67
Gabon	2012	6,067	334	15.7	53	62	52
Northern Africa		15,848	876	69.1	24	26	^*^19
Egypt	2014	15,848	876	69.1	24	26	^*^19
Southern Africa		27,823	2549	60.2	51	54	^*^46
Lesotho	2014	3,138	397	71.1	69	71	66
Namibia	2013	5,046	537	51.2	45	48	42
South Africa	2016	3,548	671	36.1	36	40	34
Zambia	2018	9,959	545	64.5	49	47	52
Zimbabwe	2015	6,132	399	68.4	57	62	^*^44
Western Africa		147,996	6099	67.9	81	89	^*^63
Benin	2018	13,589	555	61.5	70	76	^*^60
Burkina Faso	2010	15,044	573	83.2	89	96	^*^58
Cote d’Ivoire	2013	7,776	351	62.6	84	89	^*^76
Gambia	2013	8,088	281	52.3	41	37	44
Ghana	2014	5,884	427	55	46	48	44
Guinea	2018	7,951	401	71.5	87	102	^*^49
Liberia	2013	7,606	322	50.2	70	70	70
Mali	2018	9,940	345	79.9	72	78	^*^45
Niger	2012	12,558	476	86.9	81	86	^*^45
Nigeria	2018	33,924	1389	61.5	97	112	^*^73
Senegal	2018	6,719	214	62.2	40	43	36
Sierra Leone	2013	11,938	435	74.5	113	112	117
Togo	2013	6,979	330	64.2	63	70	^*^50
Central Asia		10,558	682	75.7	28	31	^*^21
Kyrgyz Rep	2012	4,363	316	70.2	26	28	23
Tajikistan	2017	6,195	366	79.1	29	32	^*^19
South-Eastern Asia		17,716	1851	68.5	26	30	^*^18
Cambodia	2014	7,165	609	85.6	29	32	*8
Philippines	2017	10,551	1242	55.9	24	27	20
Southern Asia		338,925	33064	70.9	44	49	*31
Afghanistan	2015	32,712	956	77.2	47	53	*28
Bangladesh	2014	7,886	600	74.6	41	43	35
India	2016	259,627	28332	71.9	44	49	*31
Indonesia	2017	17,848	1967	51.5	27	28	27
Maldives	2016	3,106	265	64.7	18	15	23
Nepal	2016	5,038	383	46.0	34	39	30
Pakistan	2018	12,708	561	68.1	66	72	*53
Western Asia		28,475	2050	49.3	33	43	*23
Armenia	2016	1,724	306	42.5	5	7	4
Jordan	2017	10,658	964	11.5	17	22	17
Yemen	2013	16,093	780	72.9	45	47	*38
Central America		23,328	1996	59.5	28	30	*25
Guatemala	2014	12,440	856	64.2	31	34	*26
Honduras	2011	10,888	1140	53.8	25	25	24
South America		21,379	4788	31.4	16	19	*15
Colombia	2015	11,759	3386	29.0	15	19	*13
Peru	2012	9,620	1402	34.4	17	19	16
Southern Europe		6,410	688	31.2	10	11	9
Albania	2018	2,762	652	43.9	4	7	*2
Turkey	2013	3,648	36	21.2	14	18	13
Caribbean		22,280	1863	63.1	47	51	*39
Dominican Rep	2013	3,714	518	25.7	29	33	28
Haiti	2016	6,530	450	64.9	69	71	65
Myanmar	2015	4,815	440	77.8	44	48	*30
Timor Leste	2016	7,221	455	71.3	37	41	*28
Oceania		9,514	759	89.6	40	41	32
Papua NG	2016	9,514	759	89.6	40	41	32
Total		856,987	66495	67.7	51	57	*40

*significantly different at p=0.05.

**Table 2:** Summary of pooled background characteristics of the studied children and prevalence of under-5 deaths in LMIC by rural-urban differentials in place of residence, 2010–2018.

Characteristics	Sample	%	Rural rate	Under-5 Deaths per 1000 livebirth		
				Overall	Rural	Urban
Maternal current age						
15-24	254,644	29.7	70.1	53	57	43
25-34	442,799	51.7	66.0	47	53	37
35-49	159,544	18.6	68.4	61	67	47
Maternal highest educational						
No education	292,866	34.2	83.3	69	71	59
Primary	218,432	25.5	73.7	54	55	51
Secondary+	345,689	40.3	51.3	35	39	31
Media access						
No	340,783	40.5	85.0	66	67	58
Yes	500,111	59.5	57.8	43	48	37
Maternal employment						
Employed	324,757	53.3	67.5	61	67	49
Unemployed	284,531	46.7	65.6	45	49	36
Paternal employment						
Employed	541,347	95.8	68.1	55	61	44
Unemployed	23,796	4.2	73.5	48	53	35
Marital status						
Never married	27,341	3.2	48.5	52	54	500
Currently married	791,531	92.4	68.7	51	56	38
Formerly	38,110	4.4	59.3	63	67	57
Sex of household head						
Male	718,578	83.8	68.5	52	57	39
Female	138,409	16.2	63.1	51	55	43
Wealth index combined						
Poorest	221,239	25.8	92.2	62	64	47
Poorer	193,674	22.6	85.4	58	60	46
Middle	169,849	19.8	73.2	50	53	43
Richer	148,944	17.4	50.3	45	48	42
Richest	123,281	14.4	21.0	35	35	35
Covered by health insurance						
No	671,764	87.4	70.0	55	60	44
Yes	96,784	12.6	54.8	33	38	27
Child is twin						
Single birth	834,700	97.4	67.7	47	52	37
Multiple	22,287	2.6	66.4	198	223	150
Sex of child						
Female	417,314	48.7	67.8	48	53	37
Male	439,673	51.3	67.6	55	61	43
Weight at birth						
Average+	671,296	84.0	67.6	45	50	33
Small	92,369	11.6	70.9	67	71	55
Very small	35,374	4.4	70.6	116	116	114
Birth order						
1	243,300	28.4	62.5	48	55	36
2	205,906	24.0	63.8	41	45	32
3	138,761	16.2	67.5	46	50	39
4+	269,020	31.4	76.0	66	69	56
Birth interval						
1st Birth	243,305	28.5	62.5	48	55	36
<36 months	333,066	39.0	73.6	64	69	48
36+ months	278,326	32.6	65.5	38	40	35
Drinking water						
Unimproved sources	188,610	22.7	87.2	67	68	56
Improved source	641,485	77.3	62.6	47	53	39
Toilet type						
Unimproved sources	416,964	50.3	85.4	63	64	56
Improved source	412,803	49.7	50.5	40	45	35
Cooking fuel						
Unclean/biomass	620,900	76.6	80.3	60	61	55
Clean fuel	189,870	23.4	32.5	30	32	29
Housing material						
Unimproved material	500,644	62.7	82.5	61	63	52
Improved material	298,152	37.3	48.2	41	46	37
Community SES Disadvantage						
Least	171,506	20.0	36.2	33	33	33
2	171,291	20.0	22.8	46	48	45
3	171,783	20.0	87.1	56	58	46
4	171,392	20.0	97.8	62	62	40
Highest	171,015	20.0	98.2	62	62	50
Total	856,987	100.0	67.7	51	57	40

**Fig 1:**
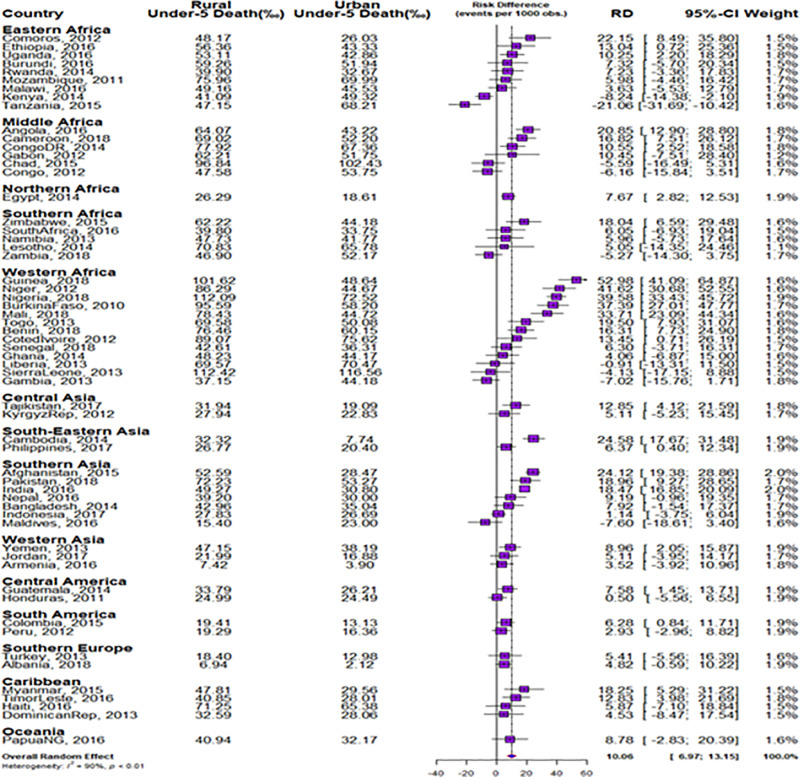
Forest plot of the risk difference in the prevalence of under-5 deaths between rural and urban children by countries.

**Fig 2:**
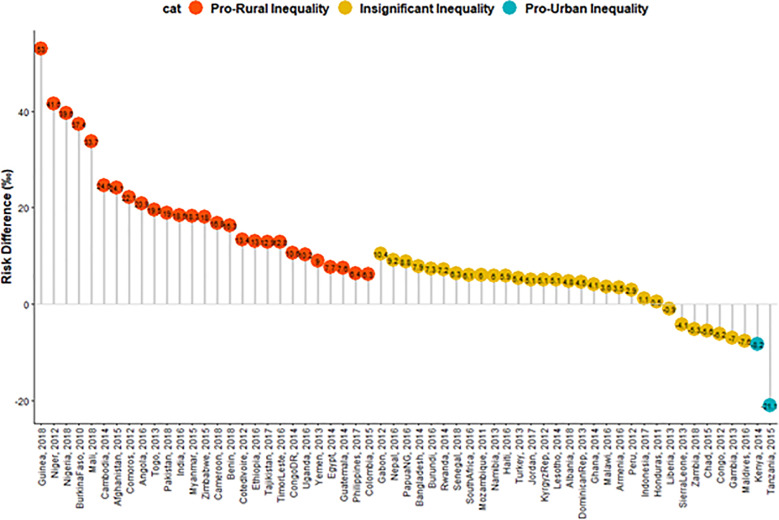
Risk difference between rural and urban children in the prevalence of under-5 deaths by countries.

**Fig 3:**
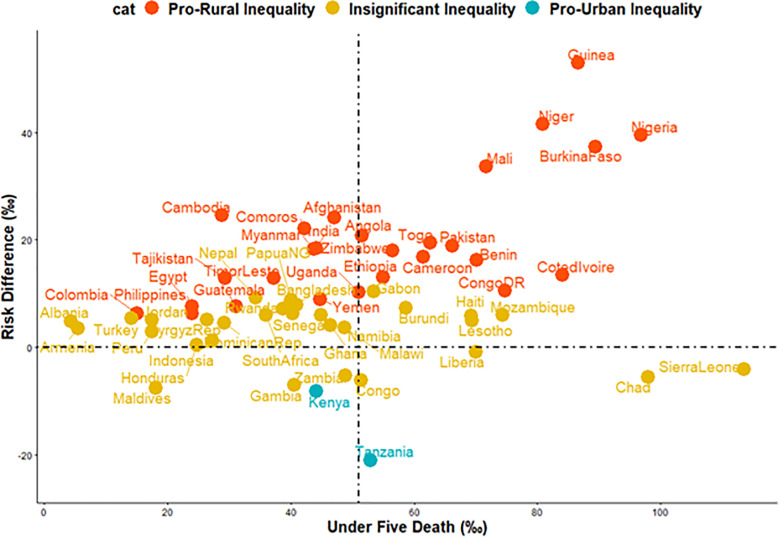
Scatter plot (and rate) of under-5 deaths and risk difference between rural and urban children in 59 countries.

**Fig 4:**
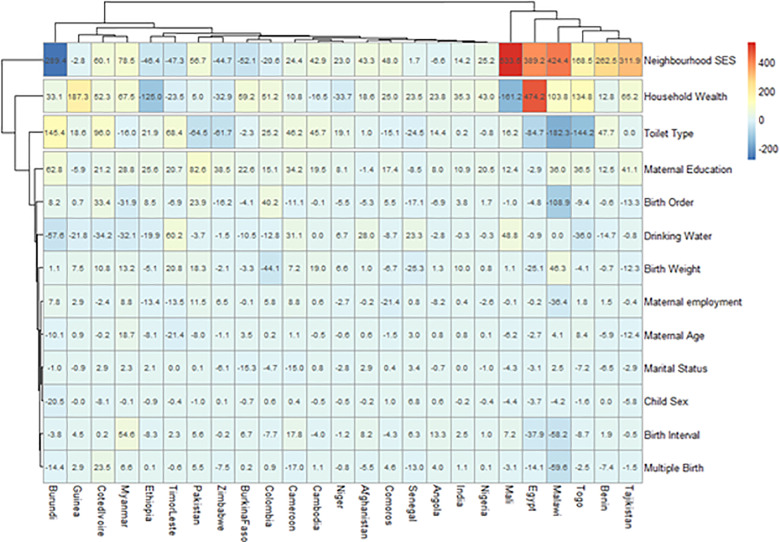
Contributions of differences in the distribution ‘compositional effect’ of the determinants of under-5 deaths to the total gap between rural and urban children among countries with pro-rural inequality.

We estimated both the random and fixed effects of the RD. The fixed effects are the weighted country-specific RD and the random effect are the overall risk difference irrespective of a child’s country (Fig 1). Charts were used to show the distributions of the RDs by the countries (Figs 2 and 3). The Mantel-Haenszel (MH) Odds Ratio (OR) and tests of heterogeneity of ORs were used to ascertain that the countries have different ORs of under-5 death among the rural and non-rural children. A test of homogeneity of ORs among all the countries with a significant OR of under-5 death was carried out. Lastly, the multivariable-adjusted binary logistic regression model was applied to pro-rural countries to carry out a decomposition analysis and the results are presented in Fig 4.

Sampling weights were applied in all our analyses to adjust for unequal cluster sizes, stratifications and to ensure that our findings adequately represent the target population.

Multicollinearity was tested using the “Colin” command in Stata version 16. The command provided the variance inflation factor (VIF). The VIF is approximate of 1/(1-*R*^*2*^) and it ranges from 1 to infinity. The *R*^2^-value is obtained by regressing the *j*^*th*^ predictor on the remaining predictors. We dropped all variables with VIF>2.5 an equivalent of *R*^*2*^=0.6. A VIF >2.5 has been reported to constitute multicollinearity [30].

Multivariable decomposition analysis is used to quantify the contributions of different risk factors to the differences in the prediction of an outcome of interest between two distinct groups in multivariate models [31]. The outputs from the regression models of group differences are partitioned into two components. The 1^st^ component is attributable to compositional differences between the two groups (endowments or explained differences) and to a 2^nd^ component which is attributable to differences in the effects of the characteristics (coefficients or unexplained differences) [31]. The Fairlie decomposition analysis (FDA) techniques were used for the multivariable analysis. The FDA is among the different decomposition techniques used in the quantification of the contributions to differences in the outcome of interest between two distinct categories [32]. The Fairlie was developed as a response to the inadequacy of the Blinder-Oaxaca Decomposition Analysis [33–35], methods for logit and probit model [36–40]. The technique constrains the predicted probability of under-5 death to between 0 and 1.

We conducted the FDA analysis was carried out by calculating the difference between the predicted probability for one group (rural children) using the other group’s (non-rural children) regression coefficients and the predicted probability for male children using its regression coefficients [37].

Fairlie et al. showed that the decomposition for a nonlinear equation $Y=F\left(X\right)$ can be expressed as in equation (1)


$${\bar{Y}}^{A}-{\bar{Y}}^{B}=|\overset{1^{st}}{\overset{︷}{\sum_{i=1}^{N^{A}}\frac{F\left(X_{i}^{A}{\hat{\beta }}^{A}\right)}{N^{A}}-|\sum_{i=1}^{N^{B}}\frac{F\left(X_{i}^{B}{\hat{\beta }}^{B}\right)}{N^{B}}}}|+|\overset{2^{nd}}{\overset{︷}{\sum_{i=1}^{N^{B}}\frac{F\left(X_{i}^{B}{\hat{\beta }}^{A}\right)}{N^{B}}-|\sum_{i=1}^{N^{B}}\frac{F\left(X_{i}^{B}{\hat{\beta }}^{B}\right)}{N^{B}}}}|$$ (1)

Where $N^{A}$ is the sample size for group *J* [41]. In equation (1), $\bar{Y}$ is not necessarily the same as $F\left(\bar{X}\hat{\beta }\right)$. The 1st part in equation (1) is the part of the gap in the binary outcome variable that is due to group differences in distributions of *X*, and the 2nd part is due to differences in the group processes determining levels of *Y*. The 2nd part captured the portion of the binary outcome variable gap due to group differences in unmeasurable or unobserved endowments.

The coefficient estimates from a logit regression model for a pooled sample, ${\hat{\beta }}^{*}$, the independent contribution of $X_{1}$ and $X_{2}$ to the group, the gap is expressed as in equations (2) and (3)


$$\frac{1}{N^{B}}X\overset{N^{B}}{\underset{i=1}{\sum }}F\left({\hat{\alpha }}^{*}+X_{1i}^{A}{\hat{\beta }}_{1}^{*}+X_{2i}^{A}{\hat{\beta }}_{2}^{*}\right)-F\left({\hat{\alpha }}^{*}+X_{1i}^{B}{\hat{\beta }}_{1}^{*}+X_{2i}^{A}{\hat{\beta }}_{2}^{*}\right)$$ (2)

and


$$\frac{1}{N^{B}}X\overset{N^{B}}{\underset{i=1}{\sum }}F\left({\hat{\alpha }}^{*}+X_{1i}^{B}{\hat{\beta }}_{1}^{*}+X_{2i}^{A}{\hat{\beta }}_{2}^{*}\right)-F\left({\hat{\alpha }}^{*}+X_{1i}^{B}{\hat{\beta }}_{1}^{*}+X_{2i}^{B}{\hat{\beta }}_{2}^{*}\right)$$ (3)

respectively. The contribution of each variable to the gap is thus equal to the change in the average predicted probability from replacing the group *B* distribution with the group *A* distribution of that variable while holding the distributions of the other variable constant [42]. Further numerical details have been reported [38,39,41,43,44].

Thus, the “Fairlie” command in STATA 16 (StataCorp, College Station, Texas, United States of America) was used to carry out the decomposition analysis to enable the quantification of how much of the gap between the “advantaged” (non-rural) and the “disadvantaged” (rural) groups is attributable to differences in under-5 deaths across the pro-rural countries [42].

## Declarations

### Ethics approval and consent to participate

We analyzed secondary data collected originally by ICF in conjunction with the participating countries. ICF had received ethical approval to conduct the study from the Institutional Review Board (IRB) of ICF Macro at Fairfax, Virginia in the USA. IRB reviewed and approved the MEASURE DHS Project Phase III. IRB in the USA.ICF granted us access and permission to use the data. There was no need for further ethical approvals. DHS Program has remained consistent with confidentiality and informed consent over the years. ICF ensures compliance with the U.S. Department of Health and Human Services regulations for the respect of human subjects. No further approval was required for this study. Further documentations on ethical issues relating to the surveys are available at dhsprogram.com

## Results

### Sample characteristics and analysis of inequality

Sixty-eight (68%) percent of the children were from rural areas, lowest (11.5%) in Jordan and the highest (90.9%) was in Burundi. The overall weighted under-5 death per 1000 children was 51, and 57 vs 40 among rural and non-rural children respectively (p<0.001). The under-5 death per 1000 children among rural children ranged from 7/1000 children in Albania and Armenia to 112/1000 each in Nigeria and Sierra Leone, while it ranged from 2/1000 in Albania to 117/1000 in Sierra Leone among non-rural children. The z-test of equality of prevalence among the rural and non-rural children was statistically significant (p<0.05) in Afghanistan (p<0.001), Albania (p<0.001), Angola (p<0.001), Benin (p<0.001), Burkina Faso (p<0.001), Cameroon (p<0.001), Comoros (p<0.001), Cambodia (p<0.001), Colombia (p<0.001), Congo DR (p<0.001), Cote d’Ivoire (p<0.001), Egypt (p<0.001), Ethiopia (p<0.001), Guatemala (p<0.001), Guinea (p<0.001), India (p<0.001), Kenya (p<0.001), Mali (p<0.001), Myanmar (p<0.001), Niger (p<0.001), Nigeria (p<0.001), Pakistan (p<0.001), Tajikistan (p<0.001), Tanzania (p<0.001), Timor-Leste (p<0.001), Togo (p<0.001), Uganda (p<0.001), Yemen (p<0.001), Zimbabwe (p<0.001).

The levels of under-5 deaths per 1000 livebirths by each category of the explanatory variables among the rural and non-rural children are presented in Table 2. The under-5 deaths per 1000 children were consistently higher among multiple births at 223/1000 children in rural areas and 150/1000 in urban areas.

### Risk differences in under-5 death among rural and non-rural under-5 children

The risk differences (RD) in having an under-5 death among rural and non-rural children across the countries studied are presented in Figs 1–3. The meta-analysis of the under-5 deaths per 1000 children among both the rural and non-rural in each of the countries are also presented in Fig 1. The under-5 deaths per 1000 children were generally higher in the rural areas than in the non-rural areas in all the countries except in Kenya, Tanzania, Chad, Congo, Zambia, Sierra Leone, Gambia, and the Maldives.

The pro-rural differences in under-5 deaths were largest in Comoros (22/1000 children) and pro-non-rural RD was widest for Tanzania (21.63/1000) in Eastern Africa. In Middle Africa, the largest pro-rural difference was in Angola (21/1000) and pro-non-rural RD was highest for Congo (6/1000). Guinea had the highest pro-rural difference in West Africa (53/1000) and the pro-non-rural difference was widest in Gambia (7/1000). In the Caribbean, the pro-rural difference was widest in Myanmar (18/1000). Irrespective of regions, the fixed effect of pro-rural differences was widest in Guinea (53/1000) while the fixed effects of pro-non-rural differences were widest in Tanzania (21/1000). Overall, the random effects, of the risk difference per 1000 children was 10.1/1000 children [95% confidence interval (CI): 7.0 -13.2], evidence of significant overall pro-rural inequality. The greatest contribution (weight) to the random effect was found in Afghanistan and India at 2% each while the least was in Comoros and Lesotho at 1.1% each (Fig 1). The overall level of heterogeneity (I^2^) in the RDs was 90% (p<0.01).

The red, orange and blue colours were used to indicate statistically significant pro-rural inequality, insignificant inequality and statistically significant pro-non-rural inequality respectively (Fig 4). A total of twenty-seven countries showed statistically significant pro-rural inequality. Kenya and Tanzania showed statistically significant pro-non-rural inequality in under-5 deaths (Figs 1–3).

### Relationship between under-5 deaths per 1000 children and magnitude of inequality

Fig 3 shows the relationships between the under-5 deaths per 1000 children and the magnitude of rural-non-rural inequality, a function of RD, across the 59 countries. We categorized the countries into four distinct categories based on their under-5 deaths per 1000 children and whether or not the differences were small or large: (i) High under-5 death prevalence and high pro-rural inequality countries such as Guinea, Niger, Mali, Nigeria and Burkina Faso (ii) High under-5 death prevalence and high pro-non-rural inequality countries such as Tanzania (iii) Low under-5 death prevalence and high pro-rural inequality countries such as Cambodia, Comoros and Afghanistan (iv) Low under-5 death prevalence and high pro-non-rural inequality countries such as Tanzania.

### Decomposition of rural-non-rural inequality in the prevalence of diarrhoea

We first computed Mantel-Haenszel pooled odds ratio (OR) estimate of having under-5 deaths while controlling for the countries among all the children. We estimated OR = 1.29 (95% CI: 1.26-1.33) and tested H_0_: OR=1. We obtained z = 22.11 and p = 0.000 and (ii) Test of heterogeneity: *X*^*2*^ = 341.57, degree of freedom (df) = 58, and p = 0.000, I^2^ (variation in odds ratio (OR) attributable to heterogeneity) = 83%. Of the 59 countries, statistically significant pro-rural odds ratio (pro-rural inequality) was recorded in only 25 countries. The countries are Afghanistan (p<0.000), Angola (p=0.001), Benin (p=0.002), Burkina Faso (p<0.001), Burundi (p=0.012), Cambodia (p<0.001), Cameroon (p<0.001), Colombia (p=0.039), Comoros (p=0.019), Cote d’Ivoire (p=0.001), Egypt (p<0.001), Ethiopia (p<0.001), Guinea (p<0.001), India (p<0.001), Malawi (p=0.033), Mali (p=0.033), Myanmar (p=0.019), Niger (p<0.001), Nigeria (p<0.001), Pakistan (p=0.010), Senegal (p=0.009), Tajikistan (p=0.004), Timor Leste (p=0.015), Togo (p<0.003), Zimbabwe (p=0.004). The Mantel-Haenszel pooled odds ratio (OR) estimate of having under-5 deaths among the children in the 25 countries while controlling for the countries was 1.48 (95% CI: 1.43-1.52) and tested the homogeneity of the ORs: *X*^*2*^ = 67.3, df. = 24, and p = 0.000.

The 25 countries with significant pro-rural inequality in the odds of having under-5 death in the Fairlie decomposition analysis. Fig 4 shows the detailed decomposition of the part of the pro-rural inequality caused by compositional effects of the determinants of under-5 deaths. The “explained” (compositional component) and the “unexplained” (structural component) portions of the rural-non-rural inequalities are depicted by red and blue colours respectively in Fig 4. The lighter the red color, the lower the percentage contribution of the “explained” portion and the lighter the blue color, the lower the percentage contribution of the “unexplained” portion.

There are large disparities in the factors associated with the pro-rural inequalities in under-5 death across the countries [42]. We identified a unique clustering among the countries as well as the factors. Tajikistan, Benin, Togo, Malawi, Egypt and Mali formed a cluster of countries where the pro-rural inequalities were explained by neighborhood SES, house wealth quintiles, toilet types, maternal education, birth order and source of drinking water. At the other side of the heat map are countries such as Burundi, Guinea, Cote d’Ivoire Myanmar, Ethiopia and Timor Leste. Generally, neighborhood SES, household wealth quintile, toilet types and maternal education, birth order and source of drinking water were the most important factors in most countries with descending contributions in that order.

Specifically, the largest contributions to pro-rural inequality in under-5 death in Tajikistan were neighborhood socioeconomic status (SES) (312% higher in communities with lowest SES), followed by household wealth quintile (65% higher among children from households in the poorest wealth quintiles), maternal education (41% higher among mothers with no education), In Mali, the greatest contributors to the disparities are neighborhood SES (534%), household wealth quintile (161%), source of drinking water (49%). The largest contributor to the inequality was neighborhood SES (424%), toilet types (182%), birth order (109%), household wealth quintile (103%), multiple births (60%) and birth interval (58%) in Malawi. Neighbourhood SES (289%), toilet types (145%), source of drinking water (58%), maternal education (63%) and household wealth quintile were the leading determinants of pro-rural inequality in under-5 deaths in Burundi.

## Discussion

This study sought to decompose characteristics associated with rural-urban inequalities in 59 LMIC across 6 continents using the Fairlie decomposition method. This study was designed to expatiate the underlying characteristics associated with under-5 mortality rural-urban inequities. Countries with significantly higher deaths in the rural area compared to the urban were regarded as having pro-rural inequities and vice versa.

In this study, we observed significant country-level variations in the risk differences between rural and urban under-5 mortalities. Within Africa, the prevalence was higher in the rural regions (pro-rural) in all countries except for Kenya, Tanzania, Chad, Congo, Zambia, Liberia, Sierra Leone, Gambia. While the Maldives alone exhibited pro-non-rural inequities associated with under-5 deaths in the whole of Asia. All other continents including America, Europe, the Caribbean, and Oceania exclusively exhibited an under-5 mortality pro-rural inequality. With growing urbanization, the importance of checking pro-non-rural inequities in under-5 mortality cannot be overemphasized. Nijman et al. argued for a need to pay close attention to urban-based dynamics that have the propensity to influence not just health indices but everything around the environment [45]. They further claimed that an overall health outcome inequality trends in this century and the requisite policies to address such inequalities can only be best understood with an in-depth understanding of urban contexts [45].

It is quite a surprising finding that more countries within Africa region compared to other regions showed pro-non-rural inequality. This may not be unconnected with slums in urban areas [46]. Undefined slum settlements within an urban region are becoming quite common in some countries today. This inadvertently puts a toll on how effective rural residents are distinguished or demarcated from their urban counterparts. Although Nigeria exhibited a highly significant pro-rural inequity of U5M in this study as supported by an earlier finding [47], we see from literature that there is an increasing number of slums interspersed in some urban areas [48]; a phenomenon that could be responsible for the pro-non-rural trend in U5M found in some African countries such as Kenya and Tanzania. Another salient factor that has been implicated in the pro-non-rural inequality of under-5 deaths in literature and validated by our study is household wealth. Considerable increase in wealth index seen in some rural areas [49] might facilitate better health-seeking practices that will ultimately lower under-5 deaths. The literature has established that household income is significantly associated with better health accessibility and affordability, lesser experiences of catastrophic health expenditure when health care access is sought [50,51].

Rural-urban inequities (RUI) have long been an item observed in several literatures which have had significant influences on different health outcomes. These health outcomes range from immunization [52], cancer mortality [53], maternal mortality [54] and resource allocation [55]. Just as in literature, our study also found a number of countries exhibiting pro-rural inequalities compared to pro-non-rural inequalities.

Factors found to be the largest contributors to pro-rural inequality were socio-economic status, maternal education, source of drinking water, toilet types, and birth orders. Our findings are corroborated by existing studies [13,20]. These factors can be grouped under maternal, social, environmental and child characteristics.

Socio-economic status is a long-standing and root cause of rural-urban health inequities. Causal pathways that explain this association has ranged from acquisition of capacities, such as accurate knowledge about health and health behaviour, to financial barriers to medical care, attitudes toward modern health services [56,57]. Semba et al. asserted that paternal smoking particularly among poor families could promote a diversion of disposable income (ideally meant for feeding) to items such as tobacco and thus promoting a higher risk of child malnutrition and all the mortalities [58].

We found that four maternal factors have direct influences on inequalities in under-5 mortality in this study – maternal education, maternal employment, maternal age and marital status. This emphasizes the importance of maternal characteristics on under-5 mortality. Policymakers need to pay close attention to maternal features when designing under-5 mortality alleviation programs. The association of maternal education with under-5 mortality as established in this study validates the literature. In other studies, emphasis is shifting to paternal education [59] which has also been found to be greater than the combined impact of income and accessibility to health services [57]. Nevertheless, the United Nations has proclaimed that education is key to improving child mortality [60].

Markedly, unemployed status, ever married or single status, female household head, insurance cover, multiple births, small weights, unimproved water sources, clean fuel were all factors that were associated with a higher risk of under-5 mortality. These are relevant issues that can be factored into survival strategies and intervention programs for maternal and child health programming.

The role of environmental factors such as housing materials has also been a long-standing factor associated with rural-urban inequities under-5 mortality. In this study, unimproved sources of water, unimproved toilet facilities, use of unclean cooking fuel and use of unimproved housing materials were all associated with under-5 mortality both in urban and rural areas. These findings corroborated existing literature [54]. Unimproved floor, wall and roof materials could expose children to insects and pollutants such as formaldehyde that can trigger undesirable health conditions in under-5 children [61]. In other studies, nutrition has been reported as an intermediate factor in the continuum of causality between poor housing and mortality [62,63]. An Indonesian study established that households that consumed more plant source foods such as fruits and vegetables, and less of grains had a greater intake of micronutrients and have a lower risk of child mortality [58]. Our study further corroborates what has been documented in the literature concerning the importance of using improved sewage and water sources if an appreciable improvement in under-5 mortality is to be achieved [3,42,64].

The importance of childbirth order, parity and childhood spacing is also very crucial in reducing rural-urban inequities in under-5 mortalities. According to a study conducted in Ethiopia, it was observed that the odds of under-5 mortality was almost 4 times higher among mothers with parity greater than six compared to parity less than four; mothers with the preceding birth interval of less than 2 years compared to those with two or more years and multiple births compared to single births [17]. A plausible explanation for this is tied to the socio-economic status of the family in which it is believed that disposable income is insufficient to attend to nutritional demands in larger families [65,66].

Besides the 39 countries that have pro-rural inequality in under-5 mortalities and consequently factored into the decomposition analysis, four countries: Kenya, Tanzania, Chad and Congo, had significant pro-non-rural inequality. This is in consonance with the literature [20]. A plausible explanation for this is the presence of slums in urban areas [42,67,68]. For instance, Nairobi, the capital city of Kenya, though highly urbanized has a large population of slum dwellers. The health conditions of children in the urban slums might be worse than those in rural areas [42,67,68]. Countries with pro-rural inequality in under-5 mortality must benchmark what is working in other countries. It would be most desirable for regions to come together to formulate policies and proffer feasible solutions that do not close gaps or reverse trends alone but also to reduce the under-5 deaths within the regions. Worldwide, there is an urgent need for policymakers to pay attention and focus on the aforementioned factors in interventions if the burden of rural-urban inequities and how they shape under-5 mortality is to be checked. Inter-regional holistic and encompassing programmatic alleviation programs are necessary to re-analyse and ensure that programs are country-specific while also maintaining focus on international implications.

### Strengths and limitations *of the* study

The data in this study was secondary. Hence, there was a limitation on the range of explanatory variables that could have been factored into the analysis. Contextual factors, political climes and environmental differences that surely differ from one continent (country or region) to another and that play crucial roles in the under-5 deaths, were not factored into this analysis. Hence some of our findings might have been under- or over-estimates. The Fairlie decomposition is an adaptation of the Oaxaca-Blinder decomposition for binary outcome variables [40]. Since these methods both cannot infer causality, we are unable to establish causation for the variables explored in this study [46]. Nevertheless, as expressly requested in literature [69], our study provides a comprehensive and in-depth analysis of factors that promote rural and urban inequality of under-5 deaths globally. Other strengths exhibited by our study lies in the use of large sample size sourced from nationally representative data that were collated across 59 countries.

## Conclusion

RUI of under-5 deaths was found to be prevalent in many of the countries examined. Study findings establish the fact that RUI found associated with under-5 deaths is not a problem that is confined to low and middle-income countries alone. According to a projection by the world bank, in order for global under-5 mortality to drop to 25 or fewer deaths per 1000 livebirths by 2030, as recommended by the Sustainable Development Goals, a projection of as low as 56.0 million deaths must be attained [5,70]. Political will needs to be rejuvenated and re-directed to the factors detailed above that currently promote the increase of under-5 deaths worldwide.
